# Vestibular Function in Patients With Vestibular Neuritis Experiencing Prodromal Dizziness

**DOI:** 10.1111/coa.14284

**Published:** 2025-01-21

**Authors:** Chisato Fujimoto, Keiko Sugasawa, Kentaro Ichijo, Mineko Oka, Teru Kamogashira, Makoto Kinoshita, Takuya Kawahara, Tatsuya Yamasoba

**Affiliations:** ^1^ Department of Otolaryngology and Head and Neck Surgery Graduate School of Medicine, The University of Tokyo Tokyo Japan; ^2^ Clinical Research Promotion Center, The University of Tokyo Hospital Tokyo Japan

**Keywords:** head impulse test, semicircular canals, vestibular evoked myogenic potentials, vestibular function tests, vestibular neuritis

## Abstract

**Introduction:**

It is unknown whether prodromal dizziness (PD) before an attack of vestibular neuritis (VN) has an association with peripheral vestibular lesions. The purpose of this study was to investigate whether the severity of vestibular dysfunction has an association with the presence of PD.

**Methods:**

We reviewed the medical records of 88 consecutive unilateral VN patients with unilateral canal paresis in caloric testing. Caloric test, cervical vestibular evoked myogenic potential test to air‐conducted sound (ACS cVEMP), ocular vestibular evoked myogenic potential test to bone‐conducted vibration (BCV oVEMP) and video head impulse test (vHIT) were used as vestibular function tests. Binomial logistic regression analyses were performed to see whether the subjects' age, sex, disease duration or the presence of PD is associated with the presence of vestibular dysfunction.

**Results:**

Seventeen (19%) experienced an episode of PD. There was no significant association between the presence of PD and abnormality in ACS cVEMPs, BCV oVEMPs, vHIT for the posterior semicircular canal (SCC) or vHIT for the lateral SCC. The presence of PD had a significant positive association with abnormality in vHIT for the anterior SCC (ASCC) (*p* = 0.0248).

**Conclusions:**

The presence of PD in VN may be associated with the peripheral vestibular lesion.

**Summary**

- The symptoms of prodromal dizziness in vestibular neuritis were all spontaneous, and most patients with prodromal dizziness reported their experience as a sensation of rotatory vertigo.
- Most episodes of prodromal dizziness occurred 1 day before the vestibular neuritis attack and were only one in number.
- There was no association between prodromal dizziness in vestibular neuritis and dysfunction of the otolith organs.
- Prodromal dizziness in vestibular neuritis had a positive association with dysfunction of the anterior semicircular canal.
- Prodromal dizziness in vestibular neuritis may be associated with the peripheral vestibular lesion.

## 1 Introduction

Vestibular neuritis (VN) is characterised as sudden rotatory vertigo lasting more than 24 h with nausea and vomiting, due to acute unilateral peripheral vestibular dysfunction, without acute central or acute auditory symptoms or signs. Recently, the Committee for the Classification of Vestibular Disorders of the Bárány Society established diagnostic criteria for acute unilateral vestibulopathy, which is synonymous with VN [1]. Acute unilateral vestibulopathy requires clear evidence of dysfunction of the vestibulo‐ocular reflex (VOR) opposite the direction of the fast phase of spontaneous nystagmus [1].

It has been previously reported that some VN patients experience transient dizziness as a prodromal symptom in the days immediately preceding the onset of a severe attack of vertigo [2, 3]. A prospective study reporting on the characteristics of prodromal dizziness (PD) before a VN attack found that approximately one in four VN patients experienced a transient episode of dizziness prior to a VN attack [2]. Reports of PD in VN are very few, and its mechanism is currently unknown. The lack of significant differences in any of the vascular risk factors such as hypertension, diabetes, and history of stroke between patients with and without PD suggests that PD is not associated with vascular events [2], and it is speculated that PD may be due to an initial smouldering inflammation of the vestibular nerve [4]. However, there is no evidence that PD has an association with peripheral vestibular lesions.

In the present study, we investigated the association between the presence of PD and the severity of vestibular dysfunction in VN patients by comparing vestibular function in groups with and without PD.

## 2 Materials and Methods

### 2.1 Research Ethics

The present study was approved by the Research Ethics Committee, Graduate School of Medicine and Faculty of Medicine, the University of Tokyo (#2487). The procedures were conducted in accordance with the tenets of the Declaration of Helsinki. Written informed consent was waived for the present study, because retrospective data from medical records were used.

### 2.2 Study Design and Subjects

We reviewed the medical records of 88 consecutive unilateral VN patients (44 males and 44 females, mean age ± standard deviation (SD) 60.9 ± 14.5 years) between January 2011 and December 2021 at the Balance Disorder Clinic of the University of Tokyo Hospital (Table 1). VN was defined as: (1) acute vertigo without auditory or neurological symptoms; (2) unilateral or bilateral abnormal findings on caloric testing; (3) no abnormal findings on magnetic resonance imaging of the brain, ear, or cerebellopontine angle; (4) no concomitant vestibular disease; (5) no other neurological disease; (6) normal hearing test or hearing loss not associated with acute vertigo.

**TABLE 1:** Comparison of demographic features in VN with or without PD.

	Total	PD group	Non‐PD group
VN patients, *n*	88	17	71
Age, mean (SD) years	60.9 (14.5)	55.2 (14.4)	62.3 (14.2)
Sex			
Male, *n* (%)	44 (50%)	8 (47%)	36 (51%)
Female, *n* (%)	44 (50%)	9 (53%)	35 (49%)
Lesion site			
Right, *n*	45 (51%)	9 (53%)	36 (51%)
Left, *n*	43 (49%)	8 (47%)	35 (49%)
Spontaneous nystagmus			
Towards the unaffected side	40 (45%)	9 (53%)	31 (44%)
Towards the affected side	3 (3%)	0 (0%)	3 (4%)
None	45 (51%)	8 (47%)	37 (52%)
Disease duration			
≤ 30 days, *n* (%)	16 (18%)	2 (12%)	14 (20%)
31–90 days, *n* (%)	32 (36%)	8 (47%)	24 (34%)
91–180 days, *n* (%)	7 (8%)	3 (18%)	4 (6%)
≥ 181 days, *n* (%)	33 (38%)	4 (24%)	29 (41%)
Vestibular function on the lesion side of VN			
ACS cVEMP, *n*	86	17	69
Normal, *n* (%)	22 (26%)	4 (24%)	18 (26%)
Abnormal, *n* (%)	64 (74%)	13 (76%)	51 (74%)
BCV oVEMP, *n*	88	17	71
Normal, *n* (%)	23 (26%)	5 (29%)	18 (25%)
Abnormal, *n* (%)	65 (74%)	12 (71%)	53 (75%)
vHIT for LSCC, *n*	60	15	45
Normal, *n* (%)	14 (23%)	4 (27%)	10 (22%)
Abnormal, *n* (%)	46 (77%)	11 (73%)	35 (78%)
vHIT for ASCC, *n*	53	13	40
Normal, *n* (%)	21 (40%)	1 (8%)	20 (50%)
Abnormal, *n* (%)	32 (60%)	12 (92%)	20 (50%)
vHIT for PSCC, *n*	53	13	40
Normal, *n* (%)	29 (55%)	6 (46%)	23 (58%)
Abnormal, *n* (%)	24 (45%)	7 (54%)	17 (42%)

Abbreviations: ACS cVEMP = cervical vestibular evoked myogenic potential to air‐conducted sound, ASCC = anterior semicircular canal, BCV oVEMP = ocular vestibular‐evoked myogenic potential to bone‐conducted vibration, LSCC = lateral semicircular canal, *n* = number of patients, PD = prodromal dizziness, PSCC = posterior semicircular canal, SD = standard deviation, vHIT = video head impulse test, VN = vestibular neuritis.

Whether the patient had prodromal dizziness was assessed by interview on the date of the first visit to the Balance Disorder Clinic, that is, the date on which the series of vestibular function tests were performed. In a previous prospective study on PD, transient dizziness that developed within 7 days prior to a VN attack was considered PD [2]. In the present study, vestibular symptoms that developed within 7 days prior to a VN attack were considered PD if they were any of those listed in the International Classification of Vestibular Disorders I, that is, vertigo, dizziness, vestibulo‐visual symptoms or postural symptoms [5].

### 2.3 Vestibular Function Tests

Caloric test, cervical vestibular evoked myogenic potential test to air‐conducted sound (ACS cVEMP), ocular vestibular evoked myogenic potential test to bone‐conducted vibration (BCV oVEMP) and video head impulse test (vHIT) were used as vestibular function tests. We used canal paresis (CP) in the caloric testing as an assessment of VOR function for the diagnosis of vestibular neuritis. Because vHIT was not introduced as a routine vestibular function test at our clinic until 2015, it was not performed on patients seen before its introduction. Caloric testing has been used to assess the function of the vestibulo‐ocular pathway relating to the lateral semicircular canal (LSCC). ACS cVEMP testing has been used to assess the function of the sacculo‐collic pathway [6, 7, 8]. BCV oVEMP testing has been used to assess the function of the utriculo‐ocular pathway [7, 9, 10]. vHIT testing has been used to assess the function of the vestibulo‐ocular pathway relating to each semicircular canal (SCC). Test procedures of each vestibular function test How to perform each test was described in Supporting Information.

### 2.4 Data Analysis

Statistical analyses were performed using EZR software version 1.55 [11]. Binomial logistic regression analyses were performed to see whether the subjects' age, sex, disease duration or the presence of PD have an association with the presence of vestibular dysfunction. The dependent variables were abnormalities in the results of ACS cVEMP, or BCV oVEMP or vHIT testing (LSCC, PSCC, ASCC), and the independent variables were age, sex (i.e., male vs. female), disease duration and the presence of PD (i.e., PD group vs. non‐PD group). Multiple regression analysis was performed to see whether the subjects' age, sex, disease duration or the presence of PD have an association with the number of vestibular end organs with dysfunction. The dependent variables were the number of vestibular end organs with dysfunction and the independent variables were age, sex, disease duration and the presence of PD. The disease duration was defined as the date of the major VN attack to the first visit to the Balance Disorder Clinic. In the first visit, a battery of vestibular function tests was performed. The four nominal groups of the disease duration were treated as dummy variables and defined as follows: if the disease duration was 30 days or less, (a1, a2, a3) = (1, 0, 0); if it was between 31 and 90 days, (a1, a2, a3) = (0, 1, 0); if it was between 91 and 180 days, (a1, a2, a3) = (0, 0, 1); if it was 181 days or more, (a1, a2, a3) = (0, 0, 0). *p* < 0.05 was considered statistically significant without adjustment for multiple testing.

## 3 Results

All 88 VN patients presented with CP on the affected side in the caloric test. Seventeen (19%) experienced an episode of PD before the onset of VN attack. Detailed demographic characteristics of VN are described in Table 1. There were eight male and nine female with the mean age ± SD at 55.2 ± 14.4 years in the PD group. The percentage of abnormal ACS cVEMP responses on the lesion side of VN was 76% in the PD group and 74% in the non‐PD group. The percentage of abnormal BCV oVEMP responses on the lesion side of VN was 71% in the PD group and 75% in the non‐PD group. The percentages of abnormal vHIT responses on the lesion side of VN in the PD group were 73% for the LSCC, 92% for the ASCC and 54% for the PSCC, whereas the percentages in the non‐PD group were 78% for the LSCC, 50% for the ASCC and 42% for the PSCC.

Symptom, onset, frequency and duration of prodromal dizziness are described in Table 2. The symptoms of PD were all spontaneous and not positional. 71% of the patients in the PD group reported their experience as a sensation of rotatory vertigo. Most episodes (65%) occurred 1 day prior to VN attack. 65% of the patients in the PD group experienced only one episode of prodromal dizziness prior to onset of VN. The most common duration of prodromal dizziness was 1–60 min (59%). On the other hand, for the nature of a major VN attack, all patients had rotatory vertigo lasting more than 24 h and only one frequency.

**TABLE 2:** Characteristics of PD in VN.

	*n* (%)
Symptoms	
Vertigo	12 (71%)
Dizziness	4 (24%)
Unsteadiness	1 (6%)
Onset of PD	
7 days before VN	1 (6%)
5 days before VN	1 (6%)
3 days before VN	2 (12%)
1 day before VN	11 (65%)
On the day of VN	2 (12%)
Frequency	
≥ 3 times	2 (12%)
Twice	4 (24%)
Once	11 (65%)
Duration	
≥ 60 min	4 (24%)
1–60 min	10 (59%)
< 1 min	3 (18%)

Abbreviations: *n* = number of patients, PD = prodromal dizziness, VN = vestibular neuritis.

First, we investigated whether the presence of PD had an association with abnormalities in VEMP testing on the lesion side of VN. As for abnormality in ACS cVEMP testing and BCV oVEMP testing, logistic regression analysis indicated that the presence of PD did not have a significant association (*p* = 0.631 for ACS cVEMP testing, *p* = 0.433 for BCV oVEMP testing) (Table 3).

**TABLE 3:** Binomial logistic regression analyses for investigating the association between the abnormalities in VEMP testing and age, sex and the presence of PD.

	ACS cVEMP		BCV oVEMP	
Variables	Odds ratio (95% CI)	*p*	Odds ratio (95% CI)	*p*
Age	1.05 (1.01–1.09)	0.0173*	1.00 (0.964–1.04)	0.992
Sex				
Male	0.533 (0.186–1.53)	0.244	0.613 (0.220–1.71)	0.349
Female	Reference		Reference	
Dummy variable (disease duration)				
a1	1.590 (0.356–7.15)	0.542	0.538 (0.132–2.02)	0.389
a2	1.170 (0.350–3.93)	0.796	0.841 (0.252–2.81)	0.779
a3	1.71 × 10^7^ (0.000–Infinity)	0.991	2.01 (0.188–21.5)	0.562
Presence of PD				
PD group	1.41 (0.348–5.69)	0.631	0.600 (0.168–2.15)	0.433
Non‐PD group	Reference		Reference	

Abbreviations: ACS cVEMP = cervical vestibular evoked myogenic potential to air‐conducted sound, BCV oVEMP = ocular vestibular evoked myogenic potential to bone‐conducted vibration, CI = confidence interval, PD = prodromal dizziness.

* *p* < 0.05.

Next, we investigated whether the presence of PD had an association with abnormalities in vHIT on the lesion side of VN. As for abnormality in the LSCC vHIT and the PSCC vHIT, logistic regression analysis indicated that the presence of PD did not have a significant association (*p* = 0.823 for the LSCC vHIT, *p* = 0.511 for the PSCC vHIT) (Table 4) On the other hand, as for abnormality in the ASCC vHIT, logistic regression analysis indicated that the presence of PD had a significant positive association (*p* = 0.0248) (Table 4). The odds ratio of abnormal vHIT at the ASCC for PD compared with non‐PD was 12.7 (95% CI, 1.38–116).

**TABLE 4:** Binomial logistic regression analyses for investigating the association between the abnormalities in vHIT and age, sex and the presence of PD.

Variables	vHIT for LSCC		vHIT for PSCC		vHIT for ASCC	
	Odds ratio (95% CI)	*p*	Odds ratio (95% CI)	*p*	Odds ratio (95% CI)	*p*
Age	1.01 (0.971–1.05)	0.615	0.991 (0.952–1.03)	0.642	0.988 (0.948–1.03)	0.560
Sex						
Male	1.06 (0.301–3.70)	0.933	1.10 (0.351–3.43)	0.873	1.13 (0.332–3.87)	0.842
Female	Reference		Reference		Reference	
Dummy variable (disease duration)						
a1	2.05 (0.191–22.0)	0.553	0.210 (0.0191–2.50)	0.221	2.96 (0.253–34.6)	0.387
a2	0.889 (0.222–3.56)	0.868	0.568 (0.141–2.29)	0.426	0.735 (0.166–3.25)	0.684
a3	2.03 (0.196–21.1)	0.552	1.11 (0.214–5.70)	0.904	0.608 (0.101–3.65)	0.586
Presence of PD						
PD group	0.850 (0.204–3.54)	0.823	1.60 (0.396–6.43)	0.511	12.7 (1.38–116)	0.0248*
Non‐PD group	Reference		Reference		Reference	

Abbreviations: ASCC = anterior semicircular canal, CI = confidence interval, LSCC = lateral semicircular canal, PD = prodromal dizziness, PSCC = posterior semicircular canal, vHIT = video head impulse test.

* *p* < 0.05.

Then, we investigated whether the presence of PD had an association with the number of vestibular end organs with dysfunction. The mean number ± SD of vestibular end organs with dysfunction was 3.77 ± 1.17 in the PD group and 3.33 ± 1.32 in the non‐PD group. Multiple regression analysis indicated that the presence of PD did not have a significant association with the vestibular end organs with dysfunction (*p* = 0.224) (Table 5).

**TABLE 5:** Multiple regression analysis for investigating the association between the number of vestibular end organs with dysfunction and age, sex, disease duration and the presence of PD.

Variables		Reference	Estimate	SE	*t*‐value	*p*
	Intercept		3.451	0.944	3.66	0.0007***
	Age		0.0014	0.013	0.10	0.918
	Sex (Female)	Male	−0.110	0.384	−0.285	0.777
Dummy variable (disease duration)	a1		−0.023	0.686	−0.034	0.973
	a2		−0.421	0.475	−0.888	0.379
	a3		−0.216	0.556	−0.390	0.699
	Presence of PD (PD group)	Non‐PD group	0.576	0.467	1.233	0.224

Abbreviations: PD = prodromal dizziness, SE = standard error.

*** *p* < 0.001.

In summary, the presence of PD had a significant positive association with abnormal vHIT at the ASCC.

## 4 Discussion

In the present study, we investigated the association between the presence of PD in VN and the results of vestibular function tests. We showed that there was no significant association between the presence of PD and abnormality in ACS cVEMPs, BCV oVEMPs, vHIT for the PSCC or vHIT for the LSCC. On the other hand, the presence of PD had a significant positive association with abnormality in vHIT for the ASCC. The presence of PD did not have a significant association with the number of vestibular end organs with dysfunction.

Considering the possibility that PD in VN may be caused by peripheral vestibular lesions, the present study examined the association between the presence or absence of PD and vestibular dysfunction. The results showed a positive association between ASCC dysfunction and the presence of PD. Although this analysis was not direct proof, the results suggest that PD is involved in the peripheral vestibular lesion, especially the ASCC lesion. The clinical significance of PD is that experiencing an episode of PD attacks prior to a significant VN attack may be a factor that exacerbates the degree of vestibular dysfunction caused by VN. On the other hand, the PD group had a higher mean number of dysfunctional vestibular end organs than the non‐PD group, but a statistically significant association between the presence of PD and the number of dysfunctional vestibular end organs was not shown. In the present study, vHIT was only performed in a subset of patients and the number of patients in whom all vestibular end organs could be assessed for function was small. Further investigation of the association between the presence of PD and vestibular function severity is needed in a larger collection of cases.

All VN cases in the present study had CP on the affected side in the caloric test and had VOR dysfunction derived from the system of the LSCC to the superior vestibular nerve. The ASCC is primarily innervated by the superior vestibular nerve, and VN patients with PD may have more severe dysfunction in the superior vestibular nerve system. However, there is no association between the presence of PD and abnormalities in the BCV oVEMPs, which reflects the function of the utricle, which is also mainly innervated by the superior vestibular nerve, and further investigation is needed to determine the effect of PD on the labyrinthine and retrolabyrinthine regions.

As for ACS cVEMPs, an association between ageing and abnormal responses was found independent of the presence of PD (Table 3). In general, it can be considered that abnormalities in VEMP testing increases with age [12, 13, 14]. Regarding the effect of ageing on morphological change in the otolith organs, it has been reported that the saccule is more affected by ageing than the utricle in terms of degeneration of hair cells and reduction of volume in the macular epithelia [15]. The ageing effect on ACS cVEMP abnormal responses should be considered apart from the presence of PD.

A previous prospective study has reported that symptoms in PD were mostly dizziness, often onset 1 day before or on the day of VN attack, only one attack, and duration from a few minutes to a few days. The present study differed from the above study in that most of the symptoms were vertigo and 35% of the patients experienced multiple attacks. The lack of significant differences in the vascular risk factors between patients with and without PD suggests that PD is not associated with vascular events [11]. However, in the present study, we observed PD with multiple recurrent attacks lasting a few minutes, and these symptoms resemble those of vascular causes, which are characterised by recurrent attacks lasting a few minutes. It is not possible to conclude that PD is not reliably associated with a vascular cause.

The present study has some limitations. First, because this is a retrospective study, there is potential for selection bias and information bias. Second, the present study is exploratory to assess whether the presence of PD is associated with the abnormality of either cVEMPs, oVEMPs or vHIT. In future confirmatory studies that focus on the association between the presence of PD and vHIT for the ASCC, adjustment for multiple testing is required to protect against erroneous conclusions. In addition, the study requires formal sample size estimation. Third, the confidence interval for the odds ratio between vHIT for the ASCC and the presence of PD was relatively wide. Fourth, cVEMP amplitudes were not corrected for SCM muscular activity, which might affect AR values. Fifth, each patient received a variety of treatments, and treatment regimens were not homogenised. It was unclear whether the presence of PD affected prognosis or treatment strategy. Sixth, even though the vestibular nerve and/or vestibular end organs are damaged to the same extent during the acute phase of VN, the results of vestibular function tests may differ depending on the degree of recovery, but there is no way to confirm this.

## 5 Conclusion

In conclusion, the presence of PD in VN may be associated with the peripheral vestibular lesion.

## Author Contributions

Chisato Fujimoto conceived of the study, conducted the experiments, analysed the data, wrote the manuscript and edited the manuscript for content. Keiko Sugasawa conducted the experiments and edited the manuscript. Mineko Oka, Kentaro Ichijo, Teru Kamogashira and Makoto Kinoshita edited the manuscript for content. Takuya Kawahara analysed the data. Tatsuya Yamasoba supervised interpretation of data and edited the manuscript for content.

## Consent

Written informed consent was waived for the present study, because retrospective data from medical records were used.

## Conflicts of Interest

The authors declare no conflicts of interest.

### Peer Review

The peer review history for this article is available at https://www.webofscience.com/api/gateway/wos/peer‐review/10.1111/coa.14284.

## Supporting information


**Data S1.** Supporting Information.

## Data Availability

The data that support the findings of this study are available from the corresponding author upon reasonable request.
