# Supplementary material for: Vestibular Function in Patients With Vestibular Neuritis Experiencing Prodromal Dizziness
Source: Clin Otolaryngol. 2025 Jan 21;50(3):507–13. doi: 10.1111/coa.14284 (PMC11975217; doi:10.1111/coa.14284)
Supplement: Supplementary file 1 — Data S1. Supporting Information. [file COA-50-507-s001.docx]

**Supplementary Methods**

**Caloric testing**

Caloric testing was performed by injecting 2 ml ice water (4 ℃) into the external auditory canal with for 20 s and recording evoked nystagmus with an electronystagmography in a dark room. This ice-water caloric testing shows a high sensitivity and specificity for the detection of CP (1). Abnormal responses were defined by either of the following criteria: (1) CP percentage > 20% for unilaterally abnormal responses (2); (2) maximum slow phase eye velocity of caloric nystagmus < 10 degrees/s for bilaterally abnormal responses (3).

**ACS cVEMP testing**

Surface electrodes were placed on the upper half of the sternocleidomastoid muscle (SCM), and reference electrodes were placed on the lateral edge of the upper sternum. During recording, subjects were supine and instructed to raise their heads from the bed to contract the SCM. 500 Hz tone burst stimuli (135dBSPL, rise/fall time 1 ms, plateau time 2 ms) were presented at 5 Hz repetition through headphones. Analysis time was 100 ms. Signals were amplified and bandpass-filtered (20–2,000 Hz) using Neuropack R (Nihon Kohden Co. Ltd., Tokyo, Japan). After confirming reproducibility with 2 runs for each ear, the amplitude and latency of the first positive–negative peak (p13–n23) ipsilateral to stimulus were determined from the average of 2 responses. p13-n23 amplitude abnormalities were evaluated by the cVEMP asymmetric ratio (AR) (4). If no reproducible p13–n23 was observed, it was judged as an absent response. The cVEMP AR greater than the normal upper limit (34.0%) was considered a decreased response (4). If there were bilateral absent responses on both sides, it was regarded as having bilaterally abnormal responses. The mean p13 latency ± SD in ACS cVEMPs in normal subjects has been reported as 14.9 ± 0.53 ms (4). The normal range of p13 was set as mean ± 2SD based on the results of normal subjects. p13 latency outside the normal range was considered as abnormal latency.

**BCV oVEMP testing**

Surface electrodes were placed on the skin 1 cm below the center of each lower eyelid, and reference electrodes were placed on the skin 3 cm below the center of each lower eyelid. During recording, subjects were instructed to look up by approximately 30 degrees. Bone conduction stimuli were provided by a 4810 mini-shaker (Bruel and Kjaer, Naerum, Denmark) placed on the forehead in the midline (Fz) with a 500 Hz tone burst (rise/fall time 1 ms, plateau time = 2 ms) with a 3 Hz repetition rate. The peak drive voltage was adjusted to 8.0 V to generate a peak force level of 128 dB (re: 1 μN). Analysis time was 50 ms. Signals were amplified and bandpass-filtered (0.5-500 Hz) using Neuropack R. After confirming reproducibility with 2 runs for each ear, the amplitude and latency of the first negative–positive peak (n1–p1) contralateral to stimulus were determined from the average of 2 responses. n1–p1 amplitude abnormalities were evaluated by the oVEMP AR (5). If no reproducible n1–p1 was observed, it was judged as an absent response. The oVEMP AR greater than the normal upper limit (27.3%) was considered a decreased response (6). If there were absent responses on both sides, it was regarded as having bilaterally abnormal responses. The mean n1 latency ± SD in BCV oVEMP s in normal subjects has been reported as 10.4 ± 0.63 ms (7). The normal range of n1 was set at mean ± 2SD on the basis of the results from normal subjects. n1 latency outside the normal range was considered as an abnormal latency.

**vHIT**

vHIT was performed using ICS Impulse (GN Otometrics, Taastrup, Denmark). Subjects were seated 1 m from a black fixation dot on a wall. The frame rate was set to 245 frames/s. Calibration based on the manufacturer’s standards was performed prior to evaluation. While the subject stared at the fixation dot, the examiner briefly and unpredictably rotated the subject’s head through a 10-to-20-degrees. The head rotations were performed in the lateral, the left anterior-right posterior and the right anterior-left posterior planes, with at least 20 repetitions in each direction, and the eye and head velocities were recorded. VOR gains were analyzed based on the manufacturer's algorithm using 175 of the total 250 samples obtained in each trial. For the measurement of the area under the curve (AUC) for head velocity, data from the start of the head movement and the subsequent zero crossing of the head velocity were used. For the measurement of the AUC for eye velocity, only the desaccaded eye data during the same interval were used (8, 9). VOR gain values were calculated as (AUC for eye velocity)/(AUC for head velocity) (8, 9). In the present study, when a mean gain in vHIT of <0.7 for the vertical SCC plane or <0.8 for the LSCC plane was detected, the relevant SCC function was regarded as abnormal

**References**

1. Schmal F, Lubben B, Weiberg K, Stoll W. The minimal ice water caloric test compared with established vestibular caloric test procedures. Journal of vestibular research : equilibrium & orientation. 2005;15(4):215-24.

2. Iwasaki S, Takai Y, Ito K, Murofushi T. Abnormal vestibular evoked myogenic potentials in the presence of normal caloric responses. Otology & neurotology : official publication of the American Otological Society, American Neurotology Society [and] European Academy of Otology and Neurotology. 2005;26(6):1196-9.

3. Fujimoto C, Murofushi T, Chihara Y, Suzuki M, Yamasoba T, Iwasaki S. Novel subtype of idiopathic bilateral vestibulopathy: bilateral absence of vestibular evoked myogenic potentials in the presence of normal caloric responses. Journal of neurology. 2009;256(9):1488-92.

4. Murofushi T, Matsuzaki M, Wu CH. Short tone burst-evoked myogenic potentials on the sternocleidomastoid muscle: are these potentials also of vestibular origin? Archives of otolaryngology--head & neck surgery. 1999;125(6):660-4.

5. Chihara Y, Iwasaki S, Ushio M, Murofushi T. Vestibular-evoked extraocular potentials by air-conducted sound: another clinical test for vestibular function. Clinical neurophysiology : official journal of the International Federation of Clinical Neurophysiology. 2007;118(12):2745-51.

6. Iwasaki S, Smulders YE, Burgess AM, McGarvie LA, Macdougall HG, Halmagyi GM, et al. Ocular vestibular evoked myogenic potentials in response to bone-conducted vibration of the midline forehead at Fz. A new indicator of unilateral otolithic loss. Audiology & neuro-otology. 2008;13(6):396-404.

7. Iwasaki S, Smulders YE, Burgess AM, McGarvie LA, Macdougall HG, Halmagyi GM, et al. Ocular vestibular evoked myogenic potentials to bone conducted vibration of the midline forehead at Fz in healthy subjects. Clinical neurophysiology : official journal of the International Federation of Clinical Neurophysiology. 2008;119(9):2135-47.

8. MacDougall HG, Weber KP, McGarvie LA, Halmagyi GM, Curthoys IS. The video head impulse test: diagnostic accuracy in peripheral vestibulopathy. Neurology. 2009;73(14):1134-41.

9. Macdougall HG, McGarvie LA, Halmagyi GM, Curthoys IS, Weber KP. The video Head Impulse Test (vHIT) detects vertical semicircular canal dysfunction. PLoS One. 2013;8(4):e61488.
